# Nucleic acid detection aboard the International Space Station by colorimetric loop‐mediated isothermal amplification (LAMP)

**DOI:** 10.1096/fba.2019-00088

**Published:** 2020-01-16

**Authors:** Julian Rubinfien, Kutay D. Atabay, Nicole M. Nichols, Nathan A. Tanner, John A. Pezza, Michelle M. Gray, Brandon M. Wagner, Jayme N. Poppin, Jordan T. Aken, Emily J. Gleason, Kevin D. Foley, David Scott Copeland, Sebastian Kraves, Ezequiel Alvarez Saavedra

**Affiliations:** ^1^ Yale University New Haven CT USA; ^2^ Whitehead Institute for Biomedical Research Massachusetts Institute of Technology Cambridge MA USA; ^3^ New England Biolabs, Inc. Ipswich MA USA; ^4^ Boeing Defense Berkeley MI USA; ^5^ miniPCR bio Cambridge MA USA

**Keywords:** diagnostics, DNA amplification, PCR, space exploration

## Abstract

Human spaceflight endeavors present an opportunity to expand our presence beyond Earth. To this end, it is crucial to understand and diagnose effects of long‐term space travel on the human body. Developing tools for targeted, on‐site detection of specific DNA sequences will allow us to establish research and diagnostics platforms that will benefit space programs. We describe a simple DNA diagnostic method that utilizes colorimetric loop‐mediated isothermal amplification (LAMP) to enable detection of a repetitive telomeric DNA sequence in as little as 30 minutes. A proof of concept assay for this method was carried out using existing hardware on the International Space Station and the results were read instantly by an astronaut through a simple color change of the reaction mixture. LAMP offers a novel platform for on‐orbit DNA‐based diagnostics that can be deployed on the International Space Station and to the broader benefit of space programs.

**Abbreviations**
ISS: International Space Station
KSC: Kennedy Space Center
LAMP: Loop‐Mediated Isothermal Amplification
MELFI: Minus Eighty Degree Laboratory Freezer
NASA: National Aeronautics and Space Administration
PCR: polymerase chain reaction

## 1 INTRODUCTION

Spaceflight is linked to a number of molecular‐, cellular‐, and systems‐level alterations in humans^1^, ^2^, ^3^, ^4^, ^5^ and other organisms, such as mice,^6^ fish,^7^ insects,^8^, ^9^, ^10^ bacteria,^11^, ^12^, ^13^ and viruses.^14^, ^15^ Moreover, different cell types in normal and disease states respond differentially to extended periods in microgravity.^16^, ^17^ To study these biological changes that impact human health during and after spaceflight, the National Aeronautics and Space Administration (NASA) and other space agencies have established several platforms on the International Space Station (ISS) to enable advanced biological research.^18^ A series of DNA analysis technologies such as the polymerase chain reaction (PCR), quantitative PCR and DNA sequencing have already been demonstrated on the ISS.^19^, ^20^, ^21^ These molecular biology technologies have contributed immensely to our understanding of the biological responses to microgravity in general. While all these efforts have been successful, they require specialized equipment and extensive molecular biology expertise to prepare, execute, and analyze the results. Additionally, completion of the experiments and interpretation of the results needs to be performed on the ground. The goal of this Genes in Space 2 investigation was to develop a simple, one‐step method to detect specific sequences of DNA aboard the ISS by a non‐specialist astronaut.

Loop‐mediated isothermal amplification (LAMP) has great potential to become an effective diagnostic tool aboard the ISS due to its unique and simple features. The target DNA sequence in LAMP is amplified at a single, constant temperature using non‐modified oligonucleotides and widely available polymerase enzymes with strand displacement activity.^22^ LAMP uses between four and six different primers to amplify a target DNA region and generates large amounts of amplified DNA product, allowing detection via a variety of simple methods.^23^, ^24^ LAMP primers recognize six to eight different regions of target DNA, and a strand‐displacing DNA polymerase starts the synthesis. One detection approach, colorimetric LAMP, takes advantage of the protons naturally released as a byproduct of DNA synthesis to acidify a weakly buffered solution and change the color of a pH‐sensitive dye visible in the reaction mixture. Starting from as little as three copies of template DNA, this change in color can be observed in as little as 20 minutes,^25^ enabling rapid and highly sensitive visual detection of target DNA amplification and making LAMP particularly suitable for the ISS as a discovery and diagnosis platform without requirement for sophisticated instrumentation.

As an initial application of LAMP, we chose to amplify telomeric DNA sequences in space. Telomeres are protective nucleoprotein caps on the ends of chromosomes that protect them from degradation and from interacting with nearby chromosomes, maintaining chromosomal stability.^26^ Aberrant regulation of telomere length has been associated with a number of human diseases and the link between physiological stress and aberrant regulation of telomere length is well documented.^27^, ^28^, ^29^ Stresses astronauts experience during spaceflight have been also shown to lead to alterations in telomere dynamics.^5^, ^30^, ^31^, ^32^, ^33^ For example, the NASA Twins Study found that the average telomere length in astronaut Scott Kelly's white blood cells increased over the course of his mission and returned to the pre‐flight length after his return to Earth.^5^ It is hypothesized that changes in telomere length during spaceflight, such as those observed in Scott Kelly, may lead to tissue‐specific molecular and cellular responses.^5^ Therefore, greater understanding of telomere dynamics in space is important for the future of human space exploration. However, studying telomeres can be challenging because telomeric DNA consists of repetitive sequences making it more difficult to amplify than, for example, a typical protein‐coding gene.^34^

Here we describe an assay that may be used to detect DNA sequences including those human telomeric repeats in as little as 30 minutes on the ISS. This assay could be adapted to monitor the length of astronauts’ telomeres during long‐duration space flight and other DNA sequences of interest.

## 2 MATERIALS AND METHODS

### 2.1 Dry run for PCR and LAMP

The miniPCR machine (mini8; miniPCR bio) was operated in the maintenance work area. A test “dry” run was executed without biological samples to confirm that the unit was operating as expected. We confirmed that the temperature profiles in this run were as expected and matched the same profiles as those on Earth (data not shown).

### 2.2 Cold stowage stability study

Complete reaction mixes were prepared for both the PCR and LAMP reactions slated for execution on the ISS. Each tube in an eight‐tube strip (Eppendorf 0030124359; Eppendorf) contained 25 μL of either Q5 Hot Start High‐Fidelity 2X Master Mix (NEB M0494; New England Biolabs, Inc) or Hot Start *Taq* 2X Master Mix (NEB M0496; New England Biolabs, Inc), along with 5 μL primers prepared at 5 μmol/L concentration, 18 μL distilled water, and 2 μL of 1 ng/μL pSPACETELO plasmid. Exact reaction conditions detailed in Table S1, primer sequences can be found in Table S2. Strips of tubes were stored at −125°C for 4 weeks and 8 weeks, thawed, and then immediately amplified. PCR amplification conditions for cold stowage stability study were as follows: 94°C/30 s [94°C/15 s, 50°C/15 s, 68°C/60 s] × 30 cycles, 68°C/2 min. LAMP amplification conditions for cold stowage stability study were as follows: 65°C/30 min. Results of cold stowage studies are shown in Table S1 and Figures S1 and S2.

### 2.3 Preparation of template DNA

The pSPACETELO plasmid template DNA was produced by inserting a 102bp sequence of human telomeric repeats (5′‐TTAGGG‐3′) into a pUC19 plasmid backbone (NEB N3041; New England Biolabs, Inc). The telomeric sequence was obtained from pBB, a plasmid obtained from Dr Jack Griffith (Addgene plasmid #53210; Addgene). Repeats were amplified from pBB using primers pBBF and pBBR (Table S3). These primers target the regions that flank the telomeric repeats. The PCR product was cleaned with a Monarch^®^ PCR DNA Cleanup Kit (NEB T1030; New England Biolabs, Inc) according to the manufacturer's instructions. The PCR product and pUC19 were digested with 20U each of two restriction enzymes: KpnI and XbaI (NEB R3142 and R0145, respectively; New England Biolabs, Inc). Following restriction enzyme digestion, the PCR product and pUC19 were gel purified and then ligated using Instant Sticky‐end Ligase Master Mix (NEB M0370; New England Biolabs, Inc). 10‐beta competent *E coli* cells (NEB C3019; New England Biolabs, Inc) were transformed and the plasmid was purified from liquid cultures of single colonies using the Monarch^®^ Plasmid Miniprep Kit (NEB T1010; New England Biolabs, Inc).

### 2.4 PCR amplification of telomeric repeats on ISS

**Figure 1:**
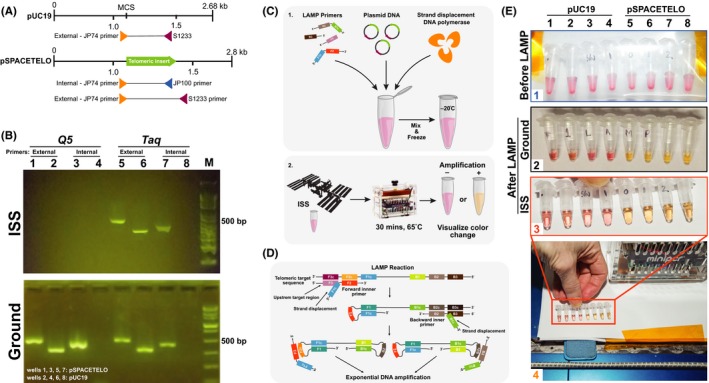
Successful amplification of target DNA by PCR and LAMP aboard ISS. A, Schematic of the plasmids used as templates for the PCR and LAMP experiments. pSPACETELO was constructed by inserting telomeric repeats, labeled “Telomeric insert” into the pUC19 vector. “External” primers JP74 and S1233 are complementary to sequences in pUC19. JP100 is complementary to a sequence within the telomeric insert. Primers pair JP74 and JP100, labeled “Internal,” will only amplify DNA if the telomeric sequence is present. B, Agarose gel electrophoresis to detect PCR products amplified on the ISS (top panel) and on ground (bottom panel). The Q5 DNA polymerase was used to amplify samples in lanes 1‐4 and *Taq* DNA polymerase was used to amplify samples in lanes 5‐8. The template DNA used in lanes 1, 3, 5, and 7 was pSPACETELO and the template in lanes 2, 4, 6, and 8 was pUC19, as indicated on the bottom panel. “External” primers were used to amplify DNA in samples corresponding to lanes 1, 2, 5, and 6 and “Internal” primers were used to amplify DNA in samples corresponding to lanes 3, 4, 7, and 8. The expected pSPACETELO amplicon using JP74 and S1233 is 503bp, and 437bp when using JP74 and JP100. The expected pUC19 amplicon using JP74 and S1233 is 350bp. No product is expected from the control reactions in lanes 4 and 8. Lane M contains fragments of DNA of known sizes ranging from 0.05 to 10.0 kilobases (1kb plus DNA ladder (NEB) in the top panel and Fast DNA ladder (NEB) in the bottom panel). C, Summary of the LAMP procedure. 1. LAMP primers, plasmid DNA, and strand‐displacing DNA polymerase were mixed in a colored, weakly buffered solution and kept frozen until the experiment was performed. 2. The samples were heated to 65°C for 30 minutes and observed for a change in color. Color change from red to yellow reflects a change in pH and indicates successful amplification of the target telomeric sequence. The experiment was done in parallel on the ISS and on the ground. D, Summary of the LAMP reaction: four primers targeting the telomeric sequence indicating strand displacement and exponential DNA amplification. Two external primers and two internal primers target the DNA sequence to generate the initial loop structures enabling subsequent rounds of DNA amplification. Amplification starts through strand invasion by one of the internal primers. A strand displacing DNA polymerase extends the internal primer. The first product is produced by DNA synthesis originating from an external primer. As the first strand is displaced, the end of the product generates a self‐hybridizing loop structure through reverse‐complementarity. This annealing and strand displacement cycle repeats on the opposite end of the target sequence. The resulting product is a dumbbell structure that serves as a seed for exponential DNA amplification. These dumbbell structures contain several sites to initiate subsequent synthesis. The outcome of these reactions is fast accumulation amplified products that allows detection of absence or a presence of a target sequence through a variety of methods. E, Colorimetric LAMP‐mediated DNA amplification detection. Template DNA is pUC19 in lanes 1‐4 and pSPACETELO in lanes 5‐8. Picture 1 shows on orbit samples prior to the experiment. Picture 2 shows samples following a 30‐minute incubation at 65°C on the ground and picture 3 the same incubation on the ISS. Picture 4 shows Astronaut Peggy Whitson holding the samples just following incubation on the ISS. A color change from red to yellow indicates that human telomeric repeats were amplified successfully. ISS, International Space Station; LAMP, loop‐mediated isothermal amplification; PCR, polymerase chain reaction

For ground samples, complete reactions were prepared as for the cold stowage studies (Table S2). Two sets of primers were used; JP74 and S1233 and JP74 and JP100 (Table S3). Primer set JP74/S1233 amplifies DNA from both empty pUC19 and pSPACETELO while primer set JP74/JP100 only amplifies DNA from pSPACETELO (Figure 1A). All primers were purchased from Integrated DNA Technologies (IDT). Amplification conditions for space samples and ground‐based controls were as follows: 94°C/30 s [94°C/15 s, 50°C/15 s, 68°C/60 s] × 30 cycles, 68°C/2 min. Following completion of the reaction, samples were stored at −80°C until their return to ground, where they were transported and later examined using electrophoresis in a 2% agarose gel with Tris/Borate/EDTA buffer at 48 volts for 30 minutes. For agarose gel analysis, fast DNA ladder (New England Biolabs, Inc) and 1kb plus DNA ladder (New England Biolabs, Inc) were used for the ground samples and ISS samples, respectively.

### 2.5 LAMP amplification of telomeric repeats on ISS

Loop‐mediated isothermal amplification primers were designed using Primer Explorer (Eiken Chemical Co., LTD) using default settings with a 1 kb region surrounding the telomere repeat of pSPACETELO as sequence input. Design of Loop primers was attempted, but only the Loop B region had sufficient space and sequence criteria for successful design and thus only Loop B primer was used in addition to the core 4 LAMP primers (Table S3). Colorimetric LAMP reactions were assembled using WarmStart Colorimetric LAMP 2X Master Mix (NEB M1800; New England Biolabs, Inc), 1.6 μmol/L FIP, 1.6 μmol/L BIP, 0.2 μmol/L F3, 0.2 μmol/L B3, and 0.4 μmol/L Loop B. Negative control (no pSPACETELO DNA) reactions were added to four tubes of the strip, and positive (pSPACETELO) to the other four, diluted final reaction volumes of 25 μL with molecular biology grade water. Reactions were stored frozen until use, then thawed and incubated at 65°C for 30 minutes. LAMP results were analyzed by visual inspection against a piece of white paper and completed reactions photographed.

### 2.6 Reaction handling and ISS operations

Polymerase chain reaction and LAMP samples were flown to the ISS aboard a Cygnus spacecraft (04.18.2017 via SS. John Glenn OA‐7) mated to a Centaur upper stage rocket and Atlas V rocket. Reactions were prepared on the ground and shipped to Kennedy Space Center (KSC) in Cape Canaveral, FL, where they were loaded into the Cygnus cargo vehicle. During the duration of on‐Earth transit, reactions were kept frozen by −20°C phase change cold packs (Cryopak). Upon successful berthing at the ISS (after 3 days of orbiting around the Earth), the tubes were transferred from Cygnus to the Minus Eighty Degree Laboratory Freezer (MELFI) in the US National Laboratory. When ISS operations commenced, astronaut crew removed the PCR and LAMP reactions from the MELFI and allowed them to thaw for 5 minutes at room temperature. Reaction parameters were programmed into the miniPCR machine using the on‐board laptop computer. After each run was complete, reaction tubes were given 30 minutes to cool down before being removed from the thermal cycler, and then transferred back to the MELFI. Tubes stayed in the MELFI until they were loaded into a SpaceX Dragon vehicle for return to ground. Upon splashdown, samples were returned to KSC in unpowered cold bags at 4°C and shipped frozen to miniPCR bio for final analysis.

## 3 RESULTS

We sought to amplify human telomeric DNA sequence in space using colorimetric LAMP and traditional PCR so that the methods could be directly compared. For both experiments, pSPACETELO, a plasmid containing a 102bp sequence of human telomeric repeats, was used as the DNA template for amplification (Figure 1A). We tested traditional PCR using both *Taq* and Q5 DNA polymerases. *Taq* DNA polymerase is routinely used for PCR amplification and is well suited for diagnostic PCRs where the readout is the presence or absence of an amplified DNA product. The use of Q5 ultra high‐fidelity DNA polymerase results in accurate replication of the template due to the enzyme's 3′ to 5′ exonuclease proofreading activity. This high fidelity is very important for next generation sequencing analysis now being performed in the ISS.^20^ LAMP reactions utilized the WarmStart Colorimetric LAMP Master Mix containing the strand‐displacing DNA polymerase *Bst* 2.0 and a formulation supporting a color change of the pH‐sensitive dye phenol red upon successful LAMP amplification. Complete reaction mixes for both the PCR and LAMP experiments were prepared prior to transport to the ISS. Prior to launch, it was determined that both the PCR and LAMP reactions were viable at temperatures as low as −125°C for up to 8 weeks, longer than the expected duration of cold stowage prior to the execution of the reaction. These tests confirmed the reactions were stable under storage conditions (Table S1 and Figures S1 and S2). For both experiments, duplicate reactions were prepared on the ground, stored under similar conditions, and performed on ground in parallel with the runs executed on board the ISS.

The PCR and LAMP reactions were launched aboard SS John Glenn OA‐7 on April 18th, 2017. The miniPCR thermal cycler used during the Genes in Space‐1 mission was also used for both Genes in Space‐2 experiments.^19^ For the PCR experiment, astronauts Jack D. Fischer and Peggy Whitson programmed the miniPCR instrument with temperatures and times for amplification that had been optimized on ground. After amplification, the PCR products were returned to ground, where the contents of each tube were analyzed by gel electrophoresis. We found that the telomeric DNA was successfully amplified in microgravity using *Taq* polymerase (Figure 1B); samples amplified in space appeared identical to those amplified on ground. DNA could only be detected in ground samples after PCR amplification using Q5 polymerase (Figure 1B). The absence of PCR product in space samples using Q5 could have several explanations, such as reduced enzyme activity due to prolonged storage or post‐amplification DNA degradation between the time of PCR and subsequent gel electrophoresis several weeks later resulting from the enzyme's 3′‐5′ exonuclease activity. Given that previous ISS experiments involving a mutant of Q5 were successful,^19^ it is unlikely that these results represent differences in enzyme activity between space and ground.

For LAMP amplification, the miniPCR instrument was used as a heat block to maintain a constant temperature of 65°C (Figure 1C). Here, we used two external primers and two internal primers targeting the telomeric DNA sequence to generate DNA loop structures enabling subsequent rounds of DNA amplification, which leads to consequent formation of diverse amplification products with multiple amplification sites for exponential DNA amplification (Figure 1D). The results of the LAMP reaction were apparent after a 30‐minute incubation; a color change from red to yellow indicated successful amplification of DNA (Figure 1E and Figure S3) in the test samples but not the control samples, indicating that human telomeric repeats were amplified successfully using the LAMP assay. Astronauts Jack D. Fischer and Peggy Whitson reported the color change to ground operations and documented the results using a consumer‐grade digital camera.

## 4 DISCUSSION

Our results indicate that telomeric repeats may be amplified in space without adaptations to the hardware or reaction conditions. This opens the door for the development of tools and diagnostic platforms that can be used broadly to detect targeted DNA sequences. We show that colorimetric LAMP is a simple and effective method to detect DNA aboard ISS with results that are apparent in real time and require minimal equipment. LAMP reagents are priced similarly to PCR master mix (eg, NEB M1800 and NEB M0494) and requires minimal equipment and astronaut time to carry out, greatly improving the overall cost‐effectiveness of the assay. In the future, assays relying on colorimetric LAMP may allow detection of clinically relevant changes in the genomes of astronauts and other life forms, as well as detection of pathogens in low‐Earth‐orbit, en route to the Moon, Mars, and beyond.

## CONFLICT OF INTEREST

EAS, EJG, and SK are employed by miniPCR bio, manufacturers of the device used for DNA amplification. NMN, NAT, and JAP are employed by New England Biolabs, Inc, manufacturer of the amplification reagents described in this manuscript. The remaining authors declare no competing financial interests.

## AUTHOR CONTRIBUTIONS

JR, KDA, NMN, NAT, JAP, EJG, SK, and EAS designed and/or performed the experiments on Earth and analyzed the data. MMG, BMW, JNP, KDF, and DSC prepared procedures for payload integration for spaceflight and oversaw operations at ISS. All authors revised and approved the manuscript. JR and KDA contributed equally to the work and are considered co‐first authors.
