# Supplementary material for: Nucleic acid detection aboard the International Space Station by colorimetric loop‐mediated isothermal amplification (LAMP)
Source: FASEB Bioadv. 2020 Jan 16;2(3):160–5. doi: 10.1096/fba.2019-00088 (PMC7059625; doi:10.1096/fba.2019-00088)
Supplement: Supplementary file 1 [file FBA2-2-160-s001.docx]

**Supplemental Materials**

**Nucleic Acid Detection aboard the International Space Station by Colorimetric Loop-Mediated Isothermal Amplification (LAMP)**

| **Identifier** | **Days at -125C** | **Days at -20C** | **Total days between sample preparation and amplification by PCR or LAMP** |
| --- | --- | --- | --- |
| #2 | 70 | 75 | 145 |
| #3 | 120 | 25 | 145 |
| #4 | 0 | 145 | 145 |

**Supplemental Table 1:** Cold stowage stability studies for PCR and LAMP samples. Identifier refers to the 8-strip tubes that were used at each of the three time points. All three samples were PCR amplified 145 days after preparation but were stored at different temperatures as indicated in the second and third columns. Refer to Supplemental Figure 1 for results of PCR amplification experiments and Supplemental Figure 2 for LAMP experiments.

**Supplemental Figure. 1. Long term stability studies for PCR assay.** Agarose gel electrophoresis to detect PCR products that had been stored according to conditions in Supplementary Table 1.The Q5 DNA polymerase was used to amplify samples in lanes 1-4 and *Taq* DNA polymerase was used to amplify samples in lanes 5-8. The template DNA used in lanes 1, 3, 5, and 7 was pSPACETELO and the template in lanes 2, 4, 6, and 8 was pUC19, as indicated above the lane numbers. 'External' primers were used to amplify DNA in samples corresponding to lanes 1, 2, 5 and 6 and 'Internal' primers were used to amplify DNA in samples corresponding to lanes 3, 4, 7 and 8 (see Figure 1a). The expected pSPACETELO amplicon using JP74 and S1233 is 503bp, and 437bp when using JP74 and JP100. The expected pUC19 amplicon using JP74 and S1233 is 350bp. No product is expected from the control reactions in lanes 4 and 8. Lane M contains fragments of DNA of known sizes ranging from 100bp to 10.0 kilobases (1kb plus DNA ladder (NEB).

**Supplemental Figure. 2. Long term stability studies for LAMP assay.** Detection of LAMP samples that had been stored according to conditions in Supplementary Table 1. Template DNA is pUC19 in lanes 1-4 and pSPACETELO in lanes 5-8. Left picture shows samples prior to the experiment. Right picture shows samples following a 30 minute incubation at 65°C. A color change from red to yellow indicates that human telomeric repeats were amplified successfully.


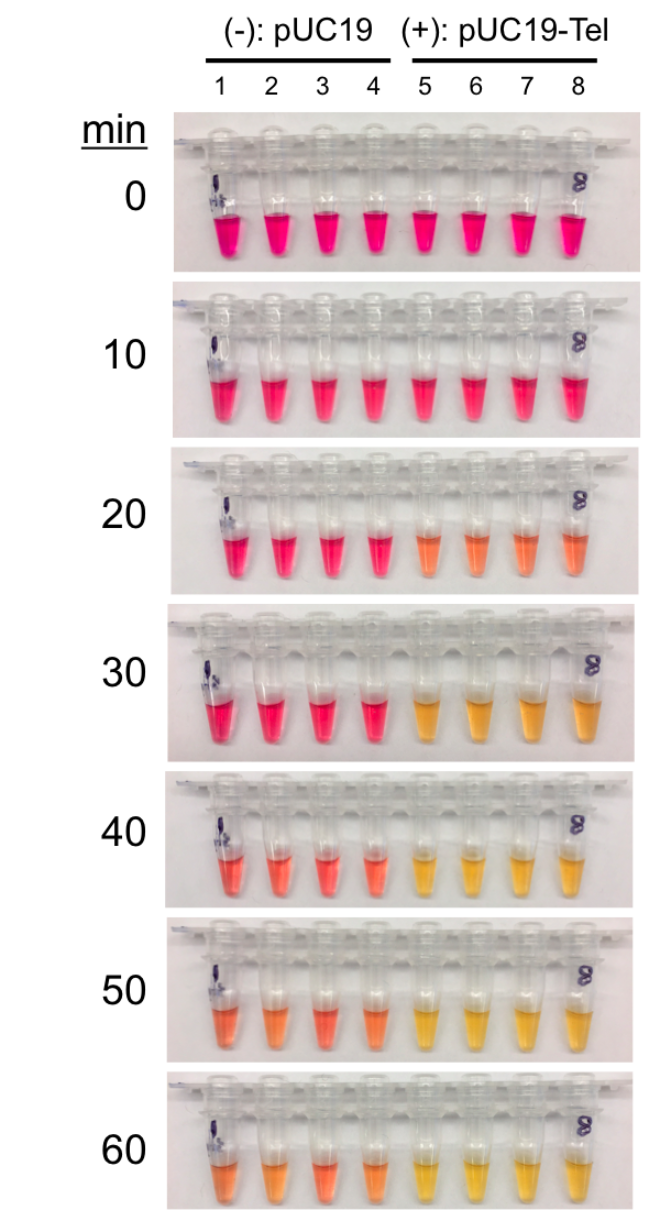


**Supplemental Figure 3: Optimization of incubation time for LAMP assay.** Samples were incubated at 65C for a total of 60 minutes. Pictures were taken every ten minutes and photographed to determine the time at which the color difference between samples that were expected to not amplify DNA (left four tubes) and to amplify (right for tubes) was most apparent. Thirty minutes was chosen as the optimal time and used in all later experiments. Template DNA is pUC19 in lanes 1-4 and pSPACETELO in lanes 5-8.

| Tube # | Polymerase | Primer Set | Plasmid Template | Expected Size (bp) |
| --- | --- | --- | --- | --- |
| 1 | Q5 | JP74 and S1233 | pSPACETELO | 503 |
| 2 | Q5 | JP74 and S1233 | pUC19 | 350 |
| 3 | Q5 | JP74 and JP100 | pSPACETELO | 437 |
| 4 | Q5 | JP74 and JP100 | pUC19 | No product |
| 5 | Taq | JP74 and S1233 | pSPACETELO | 503 |
| 6 | Taq | JP74 and S1233 | pUC19 | 350 |
| 7 | Taq | JP74 and JP100 | pSPACETELO | 437 |
| 8 | Taq | JP74 and JP100 | pUC19 | No product |

**Supplemental Table 2**: Contents of tubes used in PCR.

| Experiment | Name | Sequence |
| --- | --- | --- |
| PCR | pBBF | 5’-GTA AAA CGA CGG CCA GT-3’ |
| PCR | pBBR | 5’-CAG GAA ACA GCT ATG AC-3’ |
| PCR | JP74 | 5’-CGGCATCAGAGCAGATTGTA-3’ |
| PCR | S1233 | 5’-AGCGGATAACAATTTCACACAGGA-3’ |
| PCR | JP100 | 5’-GGCCGCTCTAGAACTAGTGGA-3’ |
| LAMP | GiS_pBBLAMPv2_F3 | 5’-GGGTTTTCCCAGTCACGA-3’ |
| LAMP | GiS_pBBLAMPv2_B3 | 5’-ACAGGAAACAGCTATGACCA-3’ |
| LAMP | GiS_pBBLAMPv2_FIP | 5’-TACCGTCGACCTCGAGGGGGGTTGTAAAACGACGGCCAGT-3’ |
| LAMP | GiS_pBBLAMPv2_BIP | 5’-AGGGTGAGACGGATCCACTAGTGATTACGCCAAGCTTGCA-3’ |
| LAMP | GiS_pBBLAMPv2_LoopB | 5’-CTAGAGTCGACCTGCAGGCA-3’ |

**Supplemental Table 3**: Primer sequences used in LAMP and PCR experiments.
